# A pilot study to evaluate the effectiveness of adjunctive use of two antimicrobial topical gels in chronic gingivitis

**DOI:** 10.4317/jced.57635

**Published:** 2021-04-01

**Authors:** Priyanka Mishra, Anuj Bhargava, Neha Nigam-Gupta

**Affiliations:** 1 Junior Resident, Department of Dentistry, Gandhi Medical College and Hamidia Hospital, Bhopal, M.P, India; 2 Associate Professor, Department of Dentistry, Gandhi Medical College and Hamidia, Hospital Bhopal, M.P, India; 3 Senior Resident, Department of Dentistry, Gandhi Medical College and Hamidia Hospital, Bhopal, M.P, India

## Abstract

**Background:**

Gingivitis is one of the most prevalent oral disease in humans. The most important etiological factor of gingivitis is dental plaque. Plaque control procedures comprises of several mechanical and chemical methods. Many studies have advocated that chemical plaque control methods can be used successfully as an adjunct to mechanical plaque control procedures. Thus, the aim of this pilot study is to evaluate the effectiveness of two topical antimicrobial gels as an adjunct to mechanical plaque control over a period of 2 weeks in treatment of chronic gingivitis patients.

**Material and Methods:**

This is a single blind, two arm parallel design pilot clinical study including 60 systemically healthy patients with 1) chronic generalized gingivitis (MGI>1), 2) probing depth ≤ 3mm and 3) zero clinical attachment loss. The study participants were randomly assigned into two groups i.e., Group I- Chlorhexidine Gluconate (CHX) gel, Group II- combination gel of Chlorhexidine gluconate and Metronidazole (CHX-MTZ) gel. Clinical parameters viz. Gingival Index (GI) and Modified Sulcus bleeding index (MSBI) were recorded firstly at baseline after Scaling and Root Planing(SRP), and secondly at the end of the study period of two weeks. Intra and inter-group comparisons of clinical parameters were done using appropriate statistical tests.

**Results:**

There was high significant reduction in GI and MSBI scores at the end of 2 weeks period in both the groups. Further, combination gel of Group II (CHX+MTZ) was found to be statistically more effective as compared to Group I (CHX) used alone.

**Conclusions:**

Our study suggests that Chemotherapeutic agents like CHX and combination CHX-MTZ Gel are clinically effective as adjunct to Scaling and Root Planning(SRP) in treatment of Chronic Gingivitis. Further, post statistical comparative analysis has proved CHX-MTZ combination gel regime to be more clinically effective than CHX gel used alone in treatment of Chronic Gingivitis as adjunct to SRP.

** Key words:**Scaling and Root Planning (SRP), Chronic Gingivitis, Chlorhexidine Gluconate (CHX) gel, Combination (CHX+MTZ) Gel, Metronidazole (MTZ).

## Introduction

Gingivitis is the inflammation of gingiva commonly characterized by redness and bleeding from gums. It is a reversible condition and also one of the most common inflammatory and prevalent disease in humans (1). It is caused by aggregation of bacterial biofilm that can be managed by either mechanical removal of this biofilm or by improving oral health status (2). Some epidemiological studies have illustrated that more than 82% of U.S. adolescents have evident gingivitis and signs of bleeding from gums. Equal or higher prevalence of gingivitis has been recorded for children and adolescents in different parts of the world (3).

One of the most important etiological factor in gingivitis is the Dental plaque which is a structurally and functionally organized biofilm (4,5). Plaque control procedures comprises of several mechanical and chemical methods. Few important mechanical modalities include SRP (Scaling and Root planning), brushing, interdental cleaning aids, flossing and dentifrices. Over a period of time, these methods have proved to be insufficient due to either being technique sensitive or dependent on the skill of the operator. Many studies were conducted to overcome these drawbacks which finally led to development of chemical plaque control methods which were introduced as an adjunct to mechanical plaque control methods (6).

The main aim of plaque control is to reduce the etiological factors causing gingivitis so as to decrease or eliminate inflammation and thereby allow healing of the gingival tissues. This can be achieved either by systemic or local administration of antimicrobial agents. A prolonged administration of systemic dose would increases the risk of problems such as antibiotic resistance and adverse drug reactions like nausea, diarrhea and pseudo membranous colitis (7). Hence, to avoid these complications, wide usage of local administration of antimicrobial agents came into existence.

CHX is widely used in the field of dentistry as antiseptics, antimicrobial and anti-plaque agent. It is available as antiseptic skin creams, topical gels, mouth rinses and also as a disinfectant to prepare the skin for surgical procedures, therefore CHX is considered as gold standard in dentistry (4). Scientific literature indicates that prolonged use of CHX leads to staining of tongue and teeth along with desquamation of intra oral mucosa or alteration in taste sensations (6). These side effects are believed to be either dose dependent or concentration dependent.

These drawbacks of CHX led to the search of its alternative. Metronidazole which is antibacterial primarily against obligate anaerobic organisms emerged as a potent drug in the treatment of gingivitis and periodontitis. Metronidazole (MTZ) antimicrobial spectrum is principally against gram positive and gram negative obligate anaerobes (4). As compared to other drugs like tetracycline, metronidazole has a narrower activity spectrum and fewer side effects and hence does not disturb the normal microbiota of the oral cavity (7).

In the current study, an attempt has been made to evaluate the effectiveness of two commercially available antimicrobial gels– Chlorhexidine Gel (Hexigel) and combination of Chlorhexidine Gluconate and Metronidazole Gel (Metrohex gel) as an adjunct to mechanical methods of plaque control over a period of two weeks in the treatment of chronic gingivitis.

## Material and Methods

Study Design: This study was a single blind, two arm parallel design pilot clinical study.

Study Population and Sample Selection: Patients were enrolled from outpatient block, of Department of Dental Surgery, Gandhi Medical College and Hamidia Hospital, Bhopal from December 2018- January 2020. Around 78 patients were assessed for the study out of which a total 60 systemically healthy patients aged between 16 - 45 years fulfilling the following inclusion criteria 1) chronic generalized gingivitis (MGI>1), 2) probing depth ≤ 3mm and 3) zero clinical attachment loss were selected for the study. Any patient with 1) A history of antibiotic intake within last three months preceding the study, 2) Pregnant or lactating women, 3) smokers, 4) chronic alcoholics, 5) known allergies to chlorhexidine gluconate or metronidazole were excluded from the study.

Data Collection Procedure: All those patients who satisfied the inclusion criteria received detailed information regarding the study and further only those patients were included who signed an informed consent ensuring their confidentiality. They were also given the option of withdrawing from the study at any given point of time without assigning any reason. A translated consent form was then completed and signed by the study participants upon agreement to participate.

Recording Clinical Parameters: The following clinical parameters were recorded: a) Gingival Index; GI as published by Loe (1967) (8). b) Modified Sulcus Bleeding Index; MSBI by A Mombelli, M.A Van Oosten, E. Schurch, Jr and N P Land, (1987) (9). A conventional manual calibrated Williams periodontal probe was used to evaluate the inclusion eligibility criteria of study subjects by assessing Clinical Attachment Level (measuring the distance between base of the pocket and the cemento-enamel junction) and Probing Depth. The validity of measurements made by the Probe to record Dental Indices was ensured by having a single trained examiner performing gentle probing of gingival margins of indexed teeth by running the probe around the teeth without application of any apical pressure and observing qualitative gingival changes to finally record GI and MSBI scores. No repeated gingival probing was done.

Methodology: Out of 78 patients, 60 patients who met the inclusion criteria were randomly assigned by simple randomization into two groups of equal sample size of 30 (thirty) each and every patient was given a code.

● Group I: 0.12% Chlorhexidine gluconate gel (Hexigel).

● Group II: combination of Chlorhexidine gluconate (0.25%) and Metronidazole (10mg) gel (Metrohex).

**Figure 1:**
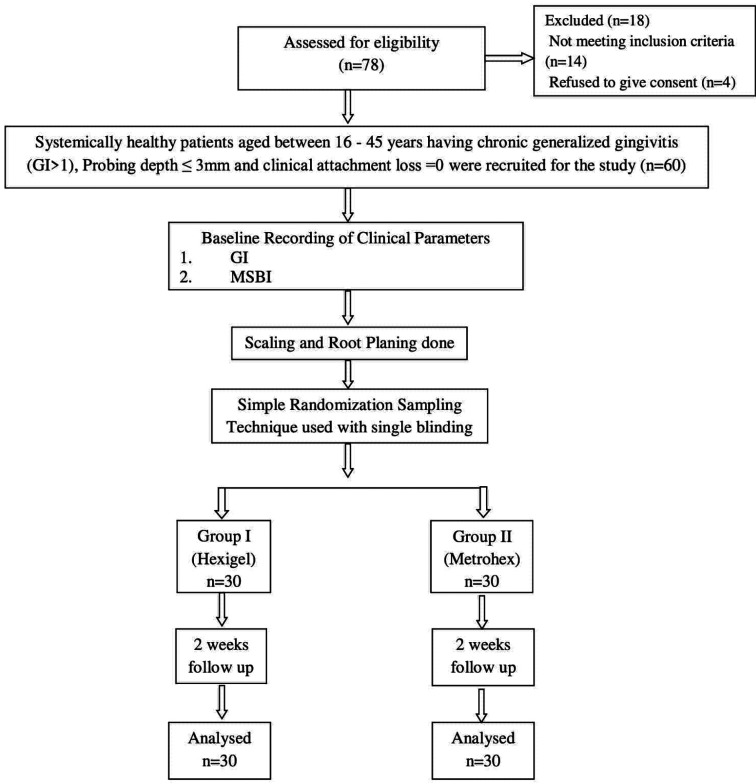
Flowchart of the study protocol showing the enrolment of subjects followed by randomization and review up to 14 days post therapy.

Hexigel (Chlorhexidine Gluconate 1% w/w) was obtained from ICPA Healthcare Products Ltd., Ankleshwar, Gujarat, India and Metrohex Gel (Metronidazole 10 milligrams + Chlorhexidine Gluconate I.P 0.25% w/w ) was procured from Dr. Reddy Laboratories Ltd., Hyderabad, India. Case History was recorded in a systematic Pro forma for the selected patients. For both Group I and II, all the clinical parameters viz. Gingival Index (GI) and Modified Sulcus Bleeding Index (MSBI) were recorded at the baseline for patients who accepted to participate in the study which was followed by SRP (Scaling and Root Planning). The antimicrobial gel tubes used in both the Groups were covered with white adhesive labels with 2 criteria’s displayed on them viz 1) Group numbers i.e. either Group I or Group II and 2) patient code. This ensured that the study subject were unaware of the gel which was used by them, ensuring single blindness of the study. Figure 1 depicts the Flowchart of the study protocol showing the enrolment of subjects followed by randomization and review up to 14 days post therapy.

After giving Phase I therapy (SRP), the patients were educated to apply a pea nut sized amount of gel allocated to them twice a day, 30 minutes after brushing and to leave it for 5minutes before rinsing. The patients were instructed to follow this regime for two weeks. No other oral hygiene instructions like flossing, inter dental aids or mouthwashes were advised to the patients. Gingival Index (GI) and Modified Sulcus Bleeding Index (MSBI) were recorded at the end of the study period i.e. 14 days (two weeks) in both Group I and II.

-Statistical Analysis:

The data obtained was subjected to statistical analysis with the consultation of a statistician. The data so obtained was compiled systematically. A master Table was prepared and the total data was subdivided and distributed meaningfully and presented as individual Tables along with graphs.

Statistical procedures were carried out in 2 steps:

1. Data compilation and presentation

2. Statistical analysis

Statistical analysis was done using Statistical Package of Social Science (SPSS Version 20; Chicago Inc., USA). Data comparison was done by applying specific statistical tests to find out the statistical significance of the comparisons. Quantitative variables were compared using mean values and qualitative variables using proportions. Unpaired student’s t-test was used for comparative evaluation between Group I and Group II using Gingival index and Modified Sulcus Bleeding Index at base line & after 2 weeks among chronic gingivitis patients. Paired student’s t-test was used for evaluation of Gingival index and Modified Sulcus Bleeding Index from base line to 2 week within Group I and Group II chronic gingivitis patients. *p* value was ascertained as: *p* > 0.05: Not significant and *p* <0.01: Highly significant (significant at 99.9% confidence level).

## Results

Table 1reveals comparative evaluation of Gingival Index between Group I and Group II at base line & after 2 week amongst chronic gingivitis patients. At baseline there was no significant difference found in gingival index value between Group I and Group II. It was 1.957±0.35 & 2.140±0.498 amongst Group I and Group II respectively. After 2 week of follow up patients oral hygiene was improved and gingival index value was significantly reduced from 1.957±0.35 to 1.535±0.343 and 2.140±0.498 to 1.049±0.383 amongst Group I and Group II respectively. It shows that Group II (Metrohex gel) is more effective as compare to Group I (Hexigel). On application of Unpaired student‘t’ test there was statistically high significant difference found in gingival index value among Group I and Group II patients after two week of application (*P*=0.001).

**Table 1:**
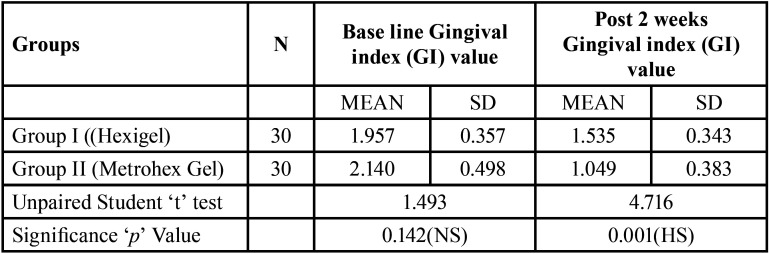
Comparative evaluation of Gingival Index between Group I (Hexigel) and Group II (Metrohex Gel) at base line & after 2 week among chronic gingivitis patients.

Table 2 reveals comparative evaluation of Modified Sulcus Bleeding Index (MSBI) between Group I and Group II at base line & after 2 week among chronic gingivitis patients. At baseline there was no significant difference found in Modified Sulcus Bleeding Index (MSBI) value between Group I and Group II. It was 1.767±0.315 &2.104±0.550 amongst Group I and Group II patients respectively. After 2 week of follow up patients oral hygiene was improved and Modified Sulcus Bleeding Index (MSBI) value was significantly reduced from 1.767±0.315 to 1.453±0.299 and 2.104±0.550 to 1.054±0.312 amongst Group I and Group II respectively. It again shows that Group II (Metrohex gel) is more effective as compared to Group I (Hexigel). On application of Unpaired student‘t’ test there was statistically high significant difference found in MSBI amongst Group I and Group II patients after two week of application (*P*=0.001).

**Table 2:**
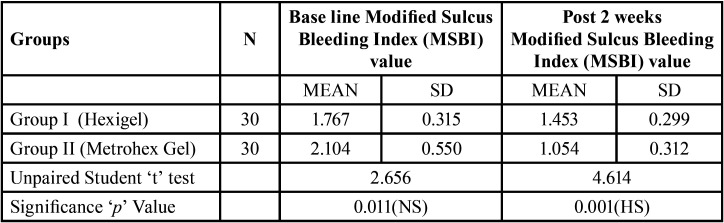
Comparative evaluation of Modified Sulcus Bleeding Index between Group I (Hexigel) and Group II (Metrohex Gel) among chronic gingivitis patients.

Table 3 reveals evaluation of gingival Index from base line to 2 week within Group I & II Chronic Gingivitis Patients. After 2 week of follow up patients oral hygiene was improved and gingival Index value was significantly reduced from 1.957±0.35 to 1.535±0.343 and 2.140±0.498 to 1.049±0.383 within Group I and Group II patients respectively. Paired student‘t’ test was applied to calculate the *p* value (*P*=0.001)

**Table 3:**
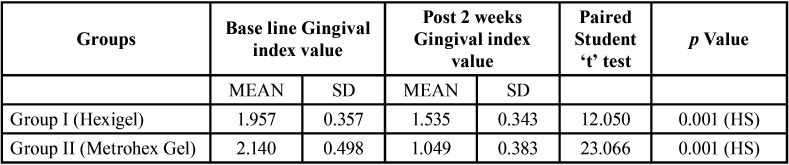
Evaluation of Gingival Index from base line to 2 weeks among Group I (Hexigel) and Group II (Metrohex Gel) among chronic gingivitis patients.

Table 4 reveals evaluation of Modified Sulcus Bleeding Index from base line to 2 week within Group I & II Chronic Gingivitis Patients. After 2 week of follow up patients oral hygiene was improved and Modified Sulcus Bleeding Index (MSBI) value was significantly reduced from 1.767±0.315 to 1.453±0.299 and 2.104±0.550 to 1.054±0.312 within Group I and Group II patients respectively. Paired student‘t’ test was applied to calculate the *p* value (*P*=0.001).

**Table 4:**
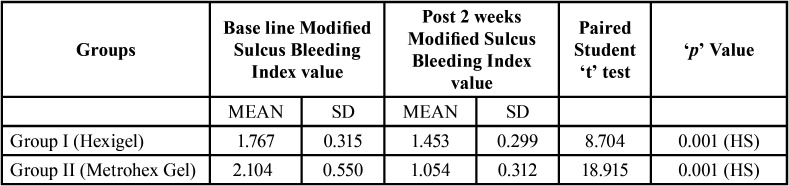
Evaluation of Modified Sulcus Bleeding Index from base line to 2 weeks among Group I (Hexigel) and Group II (Metrohex Gel) among chronic gingivitis patients.

Discussion 

The role of bacteria as an etiology in development of periodontal diseases is unarguably accepted. Periodontal diseases are chronic infections resulting from bacterial plaque deposition on tooth surfaces which lead to destruction of periodontal tissues. Bacterial plaque is a primary etiological factor responsible for inflammation of the gingival tissue. Mechanical plaque control measures of Scaling and Root Planning (SRP) have been the mainstay to control deposition of bacterial plaque. However, it is difficult to exercise absolute mechanical plaque control measures. A study conducted by Cosyn et.al discussed that the mechanical debridement procedures of SRP are adequate to reduce probing depth of gingiva and reduce bleeding on probing but a problem in reaching the base of the periodontal pockets may lead to its failure (10). Studies conducted by Griffiths GS *et al.* (2000) and Brayer WK *et al.* demonstrated that success of SRP is dependent on many factors like time spent on therapy, number of sites that require instrumentation and experience of the operator (11,12). Further, a literature search also demonstrates that there are some microflora/bacteria which are impossible to mechanically eliminate and there are also reports of such bacteria invading the peripheral periodontal tissues. Thus, some of the aforesaid shortfalls in mechanical SRP procedures lead to the quest of development of an adjunct class of chemotherapeutic agents which through a process of local drug delivery (LDD) be able to deposit higher concentrations of a drug at a target site using a low dose and causing minimal side effects as compared to systemic administration. Dr. Max Goodson in 1979 introduced the concept of LDD of a chemotherapeutic agent into localized periodontal pocket site with prolonged bioavailability and attained 100 times higher concentration of drug at subgingival target site (13). A study conducted by Chaturvedi TP *et al.* discussed the pharmacokinetics of such local drug delivery modality which provides direct access through the jugular veins and bypasses the first pass hepatic metabolism, hence leading to high bioavailability (14). In the present study an attempt has been made to evaluate the effectiveness of adjunctive use of two antimicrobial topical gels (Chlorhexidine Gel and Combination of Chlorhexidine–Metronidazole gel) administered on patients of chronic gingivitis. This study has utilized the concept of local drug delivery (LDD). Additionally a comparative evaluation of the two adjunctive drug therapies using LDD has also been done in our pilot clinical study.

Chlorhexidine (CHX) Local Drug Delivery regime is considered as a Gold Standard in Chemotherapeutic Plaque control due to its anti-plaque properties and substantivity in oral cavity. CHX has a broad spectrum of antimicrobial activity covering both Gram Positive and Gram Negative Bacteria (15). Its proposed mechanism of action includes reduction in pellicle formation, alteration of bacterial adherence to teeth, and alteration of bacterial cell wall permeability which leads to ultimately cell lysis. Commercially chlorhexidine gel is available in 1%, 0.2% and 0.12% concentrations.

Parallel to CHX another chemotherapeutic agent Metronidazole (MTZ) can be used for LDD in patients with gingivitis. Goodson (1994) reported that toxic metabolites of MTZ directly attack the bacterial cell DNA and cause cell disintegration (16). MTZ also inhibits nucleic acid synthesis, and thus produces bactericidal, anti-inflammatory & immunosuppressive effects.

Pavia et.al in their meta-analysis have shown the effectiveness of MTZ in treating Chronic Periodontistis (17). Miani *et al.* established the fact that MTZ causes reduction in bacterial count in gingival crevicular fluid (18). However, on the contrary, according to a study by Leiknes et.al MTZ does not enhance treatment result when used in combination with SRP (19).

For effective local drug delivery in treatment of gingivitis it is essential that the therapeutic agent achieves high drug concentration levels in the subgingival sites over extended periods of time. The Minimum Inhibitory Concentration (MIC) of a drug is the concentration at which it can inhibit growth of 90% strains. The MIC of Metronidazole is less than 1 mg/ml and that of chlorhexidine is 0.10 microgram/ml (20,21). In our study we have made an attempt to individually evaluate and finally compare the effectiveness/efficacy of Chlorhexidine (Hexigel) alone and combination drug Chlorhexidine-Metronidazole (Metrohex Gel) in chronic gingivitis patients using a single blinded, two arm parallel design pilot study and anatomically measuring, quantifying and statistically analyzing two Dental Indices i.e. Gingival Index(GI) and Modified Sulcular Bleeding Index(MSBI).There is a lot of literature available evaluating and comparing the effectiveness/efficacy of Chlorhexidine and Metronidazole drug therapy regimes but there is scarcity of data on evaluating the effectiveness of combination drug therapy of (CHX-MTZ) over conventional Gold Standard regime of Chlorhexidine in treatment of chronic gingivitis. Both of our study Groups I and II have used gel form of drug in order to maintain a semi solid state of drug which wouldn’t flush away by the gingival crevicular fluid. This drug state was also utilized in many other studies.

Pradeep *et al.* conducted a randomized clinical trial on 120 patients with chronic gingivitis that were randomly divided into 4 treatment groups: Group -1 Placebo gel, Group 2-Chlorhexidine (CHX) Gel, Group 3-Metronidazole (MTZ) gel, Group 4- Chlorhexidine-Metronidazole (CHX-MTZ) gel. Gingival Index (GI) of Loe and Silness and Plaque Index (PI) were used for clinical evaluation at baseline, 6 weeks, 12 weeks and 24 weeks along with microbiological assessment during the same period. The study concluded that combination gel using (CHX-MTZ) was more effective than the CHX gel which is considered a Gold standard as a chemotherapeutic modality in treatment of chronic gingivitis. The study also reported significant clinical and microbiological improvements with all the 3 treatment groups except the placebo group. Their study illustrated significant reduction of Gingival Index scores in Group-2 (CHX) and Group- 4 (CHX -MTZ) after 6 week interval and 12 week time interval and between 12 week & 24 week time interval along with reduction of microbiological counts (4). In our present study, mean Gingival Index scores for Group I (CHX) was evaluated at base line post SRP and after 2 weeks. A high degree of significant reduction in Gingival index value was obtained just after 2 weeks from baseline within this Group. Thus, the reduction in GI scores after application of CHX gel is consistent with the aforesaid study. Similarly the combination drug (CHX-MTZ) Group II of our study showed significant reduction in GI scores after 2 weeks which are also well consistent with the referred study. An overall comparative evaluation between Group I (CHX) and Group II (CHX-MTZ: Combination Drug) showed high statistical significant reduction in Gingival Index Scores in Group II as compared to Group I. Further, in our study, statistically high significant reduction in Modified Sulcus Bleeding Index from base line after 2 weeks was recorded within Group I (CHX) and Group II(CHX-MTZ) but an overall comparative statistical evaluation between Group I (CHX) and Group II (CHX-MTZ: Combination Drug) revealed high significant reduction in Modified Sulcus Bleeding Index scores in Group II as compared to Group I. Statistically Significant reduction in GI and MSBI scores in Group I (CHX) is attribuTable to the anti-plaque effect of CHX which has been effectively used since long time in therapeutic management of gingivitis and periodontitis along with SRP. These results are consistent with the study results of Fine JB *et al.* who concluded that irrigation of subgingival tissue with CHX caused significant lowering of supragingival plaque and bleeding on probing as compared to the control group (22). The overall comparative evaluation between Group I (CHX) and Group II (CHX-MTZ) statistically reported high significant reduction in Group II as compared to Group I for both GI and MSBI Scores. Thus, our study results are strongly indicative to the fact that CHX-MTZ gel combination drug regime, having the conjoined properties of both components is more efficacious than CHX gel used alone in chronic Gingivitis patients. These findings are well consistent with the aforesaid study of Pradeep et.al as well. However, the time period of assessment in our pilot study was short (Only 2 weeks) and not in congruence with the above reported study which evaluated the patients up to 24 weeks from baseline. In the present study highly significant and statistically reducing trend of GI & MSBI index scores were obtained just after 2 weeks in both Group I and II along with high significant reduction in Group II as compared to Group I. The results are encouraging but definitely require long term evaluation and larger sample size. No microbiological analysis was done in our present study.

The study conducted on special patients by Pannuti *et al.* illustrated that use of 0.5% CHX gel was effective in causing reduction in gingival bleeding in special patients as compared to the placebo group (23). Statistically in our study also there was a high significant reduction in Modified Sulcular Bleeding scores of patients with gingivitis after 2 weeks. In simple words after application of CHX gel in Group I of our study, redusced gingival bleeding was observed in patients after 14 days.

Vibha Singh *et al.* conducted a comparative evaluation of topical application of turmeric gel and 0.2% Chlorhexidine Gluconate gel in prevention of gingivitis in a sample size of 40 subjects between age group of 25-35 years. Gingival,Plaque index and Sulcular bleeding index were recorded at 0, 14 and 21 days. Statistical analysis of data concluded that both turmeric gel and chlorhexidine gel were efficacious adjuncts to mechanical plaque control in prevention of gingivitis. However, Chlorhexidine gel was found to be more effective when anti-plaque and anti-inflammatory properties were observed (6). The above study indicates that Chlorhexidine gel possess higher efficacy as compared to other local chemotherapeutic drug in reduction of gingivitis. Similarly, in the present study intra Group I (CHX) analysis showed effective and statistically significant reduced Gingival and modified sulcular bleeding index score after 2 weeks from baseline. Although, when a comparative evaluation between Group I (CHX) and combination drug Group II (CHX-MTZ) was done, Group II reported high statistical significance indicating CHX-MTZ combination to be more effective and efficacious than CHX. This may be attributed to the synergistic substantive property of CHX coupled with bactericidal potential of MTZ bundled together.

However, we feel that there are certain limitations in the present study which are as follows: - This is a two arm parallel design, single blind, pilot clinical study with a small sample size. The time period for evaluating the clinical parameters may be increased along with incorporation of microbiological analysis in future studies with additional diagnostic aids. Further, long term prospective clinical studies need to be carried out for detailed evaluation and proposing a conclusive remark over the efficacy & effectiveness of combination formulae (CHX-MTZ) over and above gold standard regime of CHX in treatment of Gingivitis and Periodontitis.

## Conclusions

CHX as an anti-plaque agent has proved to be a gold standard amongst various topical local drug delivery regimes used in treatment of Chronic Gingivitis as an adjunctive to mechanical plaque control measures. The present pilot study suggests that Chemotherapeutic agents like CHX and combination CHX-MTZ Gel are efficacious and clinically effective as adjunct to Scaling and Root Planning (SRP) in treatment of Chronic Gingivitis as evident from the reduced GI and MSBI scores post 2 weeks from baseline. Further, post statistical comparative evaluation and analysis between the two antimicrobial agents used in the study as adjunct to SRP, CHX-MTZ combination gel regime has proved to be more clinically effective than CHX gel used alone in treatment of Chronic Gingivitis.
